# Effect of Waste Tire Particle Content on the Compressive Behavior and Pore Structure of Loess Subgrade Materials

**DOI:** 10.3390/ma18225078

**Published:** 2025-11-07

**Authors:** Xueyu Cao, Yang Liu, Xun Wu, Meng Han, Xiaoyan Liu

**Affiliations:** 1 School of Highway, Henan College of Transportation, Zhengzhou 451460, China; caoxueyu2004@163.com; 2 School of Mechanics and Civil Engineering, China University of Mining and Technology, Xuzhou 221116, China; 15290857744@163.com; 3 Department of Architectural Engineering, Henan College of Transportation, Zhengzhou 451460, China; 4 College of Civil and Transportation Engineering, Hohai University, Nanjing 210098, China; 5 School of Civil Engineering, Dalian University of Technology, Dalian 116024, China; hanmeng320@mail.dlut.edu.cn

**Keywords:** waste tires, loess, tire-derived aggregate-loess mixture (TDA-LM), consolidation test, void ratio, compression characteristics

## Abstract

In response to the challenges of low recycling rates of waste tires and their underutilization in loess subgrades, this study systematically investigates the compression deformation characteristics of tire particle (4–6 mm)-modified loess through comprehensive laboratory testing. Using one-dimensional compression tests and cyclic loading–unloading tests, the effects of different tire particle contents (0% to 100%) on pore structure evolution, compression parameters—including the compression coefficient, compression modulus, and volumetric compression coefficient—and deformation mechanisms were thoroughly analyzed. The study reveals critical state characteristics and deformation mechanisms of tire-derived aggregate–loess mixtures (TDA-LMs) and establishes a predictive model for their compression behavior. The research results indicate the following: (1) The compression behavior of TDA-LM exhibits a distinct dosage threshold and stress dependence: the critical blending ratio is 30% under stresses below 100 kPa, increasing to 40% at higher stresses (≥100 kPa); (2) Mixtures with medium to low tire content display strain hardening, whereas pure tire specimens show approximately 10% modulus softening within the 200–300 kPa range. Stress- and content-dependent models for the compression modulus and volumetric compression coefficient were developed with high accuracy (*R*^2^ > 0.96); (3) The dominant deformation mechanism shifts from soil skeleton plastic yielding (at tire contents < 40%) to rubber-dominated elastic deformation (at contents > 50%). Over 85% of cumulative deformation occurs during the initial loading phase, indicating that particle–soil interface restructuring primarily takes place early in the loading process. This study provides a theoretical basis and practical design parameters for the application of waste tires in loess subgrade engineering, supporting the sustainable reuse of solid waste in environmentally friendly geotechnical construction.

## 1. Introduction

With the rapid expansion of China’s automotive industry and the continuous rise in residential living standards, the number of motor vehicles in operation has grown exponentially [1,2,3]. According to the most recent data from the Traffic Management Bureau of the Ministry of Public Security, China’s motor vehicle fleet reached 453 million units by the end of 2024, which included 353 million passenger cars and commercial vehicles [4,5,6]. This surge has directly led to the generation of a substantial volume of end-of-life tires. As estimated by the China Rubber Industry Association (CRIA), more than 20 million waste tires are produced annually in the country, with a total weight of approximately 15 million metric tons and an annual growth rate of 8–10% [7]. Improper disposal of these tires not only consumes considerable land resources but also increases the risk of fires and contributes to “black pollution,” posing serious threats to ecological integrity and public health [8,9,10]. Under conventional treatment approaches, the main disposal pathways for waste tires currently include retreading (around 5%), recycled rubber production (about 30%), rubber powder manufacturing (roughly 15%), and energy recovery (approximately 20%). Nonetheless, nearly 30% of waste tires still remain underutilized [11,12]. Therefore, developing new pathways for recycling waste tires is of considerable practical importance.

Simultaneously, large-scale infrastructure development is ongoing in China’s Loess Plateau region. Loess, a distinctive Quaternary sediment widely distributed across northwestern China, covers an area of approximately 630,000 square kilometers [13,14,15]. In engineering practice, loess often displays adverse characteristics such as collapsibility upon wetting and structural instability, necessitating ground improvement [16,17,18]. Conventional methods like lime stabilization and cement solidification, though effective, are associated with high costs, significant energy consumption, and substantial carbon emissions [19,20,21]. The use of waste tire particles for loess improvement offers a promising green technology that delivers dual advantages: (1) enabling the resource recovery of solid waste and (2) enhancing geotechnical properties, while addressing pressing environmental and engineering challenges [5,6].

Research on the compression characteristics of tire particle-modified soils has evolved progressively from sand-dominated systems to specialized soil types, reflecting substantial advances in both fundamental mechanisms and practical applications. Early investigations [22,23,24] established a foundational framework for the mechanical behavior of tire particle–sand mixtures, identifying two key phenomena within the 10–40% blending ratio range: (1) significant strength enhancement and (2) a notable increase in compressibility beyond the 30% threshold. Kim and Santamarina [25] elucidated this phenomenon at the microscale using X-ray computed tomography (CT), revealing a critical microstructural transition: below 30% rubber content, the sand matrix retained the load-bearing skeleton, whereas beyond 60%, a continuous rubber network formed. This structural reorganization directly accounted for the abrupt shift in macroscopic compression behavior. Ghazavi and Sakhi [26] further identified a critical particle size effect in tire-derived aggregate–sand mixtures (TDA-SMs), showing that normal stress, sand matrix unit weight, shred content, shred width, and the aspect ratio of tire shreds are the key parameters influencing the shear strength characteristics of sand–shred mixtures. Through systematic experimentation, Bali Reddy et al. [27] determined that the optimal rubber content range lay between 30 and 40% for such mixtures—a finding especially relevant to loess improvement engineering. Collectively, these studies demonstrate that the compression behavior of TDA-SM was governed by three principal factors: (1) blending ratio, (2) particle geometry (size and shape), and (3) soil type and structure. Recent research has increasingly focused on developing advanced constitutive models. Mashiri et al. [28,29] contributed significantly by formulating an elastoplastic model incorporating state parameters to accurately capture compression behavior within the transitional blending ratio range (30–40%). Their proposed shear modulus degradation function has become a key reference in engineering practice. Notably, Lv et al. [30] employed the Discrete Element Method (DEM) to investigate the micromechanical behavior of TDA-SM, revealing that particle orientation plays a crucial role in controlling shear band development and volumetric response.

Building upon insights from TDA-SM research, the application of waste tires has been expanded to a variety of soil types—including clay, expansive soil, soft soil, and loess—each demonstrating distinct improvement effects tailored to their specific geotechnical characteristics. The optimal improvement strategies vary considerably across soil types, depending on inherent properties and engineering requirements. In cohesive soils, Akbulut et al. [31] attributed enhanced compression properties to the incorporation of rubber, where its elastic deformation mitigates the plastic yielding of clay. Cetin et al. [32] determined that the optimal content of coarse-grained and fine-grained tire particles in clay was 20% and 30%, respectively—a difference arising from particle-size-dependent compatibility with the porous structure of clay. For expansive soil, Yang et al. [33] investigated the physical and mechanical properties of waste tire-improved samples and found that a 20% rubber content not only increased stability and strength but also significantly enhanced expansion, shrinkage, and consolidation characteristics. In soft soil improvement, Ajmera et al. [34] identified a critical coupling effect between particle size and content, showing that the addition of 2–4% rubber particles increased the unconfined compressive strength by 35–45%. This improvement was attributed to two synergistic mechanisms: (1) granular filling and (2) interfacial reinforcement. Regarding loess improvement, Li and Zhang [35,36] and Li et al. [37] systematically studied the influence of rubber content on compressive and shear behavior, identifying the 30–40% range as optimal for balancing compressibility and strength. In an innovative approach, Chen et al. [38] combined waste tire particles with Enzyme-Induced Carbonate Precipitation (EICP) technology for loess stabilization, achieving a 50% increase in shear strength. The optimal parameters were identified as 1–2 mm particle size and approximately 10% content.

However, current research remains predominantly focused on conventional soils such as sands and clays, while systematic studies on special soil types—particularly loess—are still limited. Moreover, the understanding of interaction mechanisms between tire particles and soil matrices remains superficial, lacking comprehensive multiscale mechanistic insights. Additionally, existing studies have not adequately addressed the stress-dependent evolution of the mechanical behavior of modified soils, resulting in limited guidance for practical engineering applications. This is particularly evident in the absence of standardized methodologies for determining critical blending ratios. To address these gaps, this study investigates the critical state characteristics of tire particle-modified loess under various stress levels and identifies the critical admixture ratio for optimal improvement. The evolution mechanisms of compression parameters were examined, with emphasis on how tire particle content influences the compression coefficient, compression modulus, and volume compressibility. Further, the distribution of elastic–plastic deformation under cyclic loading-unloading conditions was analyzed, and differences in deformation contributions between initial loading and subsequent cycles were clarified. This research provides a theoretical basis and technical parameters for the resource utilization of waste tire particles in loess subgrade engineering.

## 2. Materials and Methods

**Figure 1:**
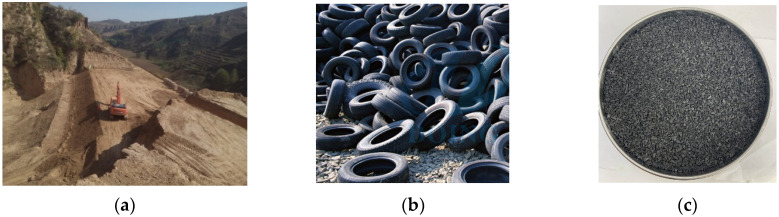
Test materials: (**a**) loess on the Yan’an Expressway; (**b**) waste tires; (**c**) waste tire particles.

**Figure 2:**
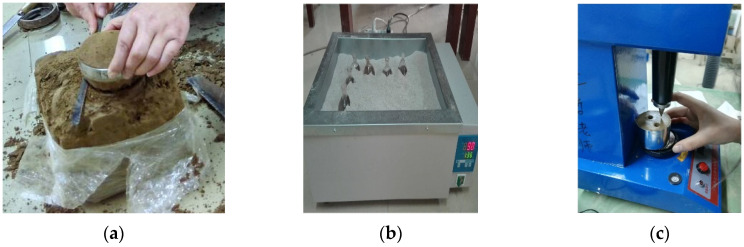
Main experimental equipment: (**a**) cutting ring sampling; (**b**) sand bath setup; (**c**) liquid–plastic limit combined tester.

**Figure 3:**
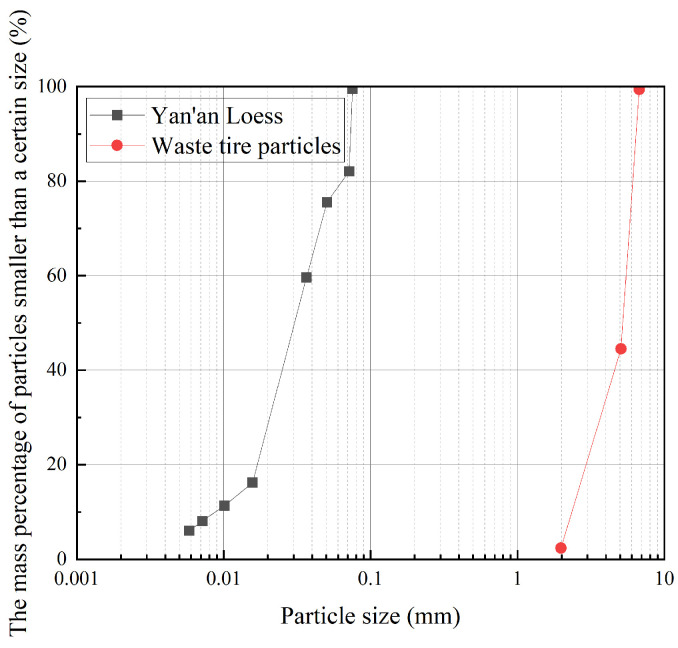
Particle size distribution curves of loess and waste tire particles.

Loess sampled from the roadbed slope of the Yan’an Expressway in Shaanxi Province, and waste tire particles were selected as the primary experimental materials. The waste tire material, sourced from passenger car tires, was processed by removing steel belt reinforcements and manually cutting into equidimensional particles with a side length of 4–6 mm (see Figure 1). In accordance with the Chinese “Standard for Geotechnical Testing Method” (GB/T 50123-2019) [39], the density, water content, and specific gravity of the loess samples were determined using the cutting ring method, oven-drying method, and pycnometer method, respectively. The liquid and plastic limits were measured with a digital liquid–plastic limit combined tester. The digital liquid–plastic limit combined tester (Model: LP-100D) used in this study was manufactured by Hebei Guanghui Testing Instrument Co., Ltd. (Cangzhou City, China), with a resolution of 0.1 mm and a maximum measuring range of 22 mm. Figure 2 presents the main experimental equipment used for assessing the physical and mechanical properties of the loess, and Table 1 summarizes these measured properties. The particle size distribution of the loess was determined via the hydrometer method, while that of the tire-derived particles was obtained through sieve analysis. The resulting particle size distributions of both materials are shown in Figure 3. Based on the American ASTM D6270-20 standard [40] and the particle size testing results, the manually cut tire particles were classified as granulated rubber.

This study focused on mixtures of loess obtained from the Yan’an Expressway slope and waste tire particles, with the primary objective of investigating the effect of varying tire particle content on the compression performance of the improved loess. The experimental design followed the methodology established by Li and Zhang [35,36], incorporating eight blending ratios: 0% (pure loess control), 10%, 20%, 30%, 40%, 50%, 75%, and 100% (pure tire particles control). The specimen preparation process consisted of three stages: (1) determination of the optimum moisture content for each mixture via light compaction tests in accordance with ASTM D6270-20 [40]; (2) fabrication of samples at the target dry density and optimum moisture content, involving calibrated water addition and mechanical homogenization; (3) sealed curing for 24 h to ensure uniform moisture distribution. The physical properties of the prepared specimens are summarized in Table 2. The initial void ratio of each sample was calculated using Equation (1):

(1) $$e_{0}=\frac{\rho_{w}G_{s}(1+0.01w_{0})}{\rho_{0}}-1$$

where $e_{0}$ was initial void ratio; $\rho_{w}$ was density of water; $\rho_{0}$ was initial density; $G_{s}$ was specific gravity of loess; $w_{0}$ was initial water content.

**Figure 4:**
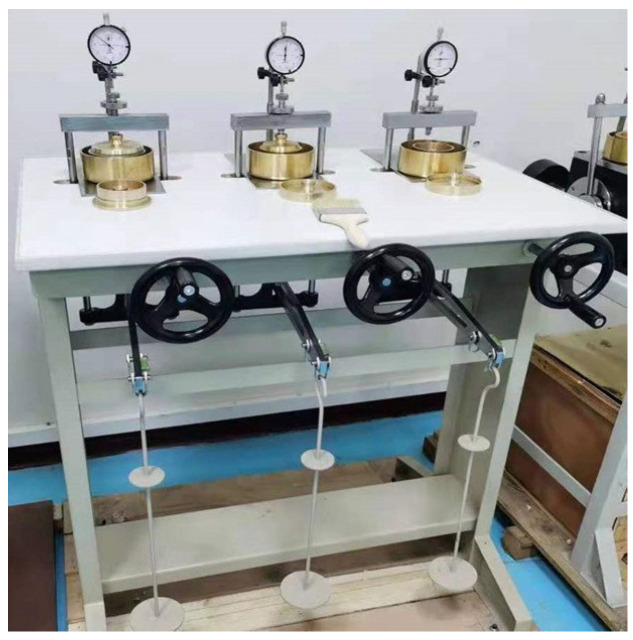
Consolidation tester.

According to the “Standard for Geotechnical Testing Method” (GB/T 50123-2019) and the “Specification of Soil Test” (SL 237-1999) [41], one-dimensional consolidation tests were performed using a WG single-lever consolidometer manufactured by Nanjing Ningxi Soil Instrument Co., Ltd. (Nanjing, China). The apparatus offers dual loading configurations with pressure ranges of 12.5–1600 kPa (for a 30 cm^2^ platen) and 12.5–800 kPa (for a 50 cm^2^ platen), as shown in Figure 4. In accordance with GB/T 50123-2019, the applied incremental loads were set at 12.5 kPa, 25 kPa, 50 kPa, 100 kPa, 200 kPa, 300 kPa, and 400 kPa. A maximum load of 300 kPa was applied to low-dosage specimens (0–50% dosage of tire particles), governed by the soil skeleton, to reach their critical state without failure, whereas a 400 kPa load was necessary for high-dosage specimens (75–100% dosage of tire particles), governed by the tire skeleton, to fully mobilize their mechanical response and observe failure mechanisms. The testing procedure consisted of the following steps: (1) initial loading up to 300–400 kPa; (2) stepwise unloading back to 12.5 kPa using the same stabilization criteria; (3) a second full loading-unloading cycle. When settlement rate measurement was not required, the stabilization criterion for each pressure stage was defined as either (1) consolidation for 24 h or (2) a specimen deformation rate not exceeding 0.01 mm per hour. After recording stable readings at each pressure level, the next load was applied progressively until the test was completed.

## 3. Results

According to the “Standard for Geotechnical Testing Method” (GB/T50123-2019), the void ratio of the specimen after stabilization under graded loads of various levels is calculated using Equation (2):

(2) $$e_{i}=e_{0}-\frac{1+e_{0}}{h_{0}}\sum \Delta h_{i}$$

where $e_{i}$ was the void ratio under a certain pressure; $e_{0}$ was the initial void ratio; $h_{0}$ was the initial specimen height; $\Delta h_{i}$ was deformation under the current load.

The compression coefficient for a specified pressure range is calculated using Equation (3):

(3) $$a_{v}=\frac{e_{i}-e_{i+1}}{p_{i+1}-p_{i}}\times 10^{3}$$

where $a_{v}$ was the compression coefficient; $p_{i}$ was a given unit pressure value.

The compression modulus and volumetric compression coefficient for a specified pressure range are calculated using Equations (4) and (5), respectively:

(4) $$E_{s}=\frac{1+e_{0}}{a_{v}}$$

(5) $$m_{v}=\frac{1}{E_{s}}=\frac{a_{v}}{1+e_{0}}$$

where $E_{s}$ was the compression modulus; $m_{v}$ was the volumetric compression coefficient.

The void ratio, compression coefficient, and compression modulus tests were calculated using Equations (2)–(5) (see Table 3).

### 3.1. Effect of Different Proportions of Waste Tire Particles on the Void Ratio of Loess

**Figure 5:**
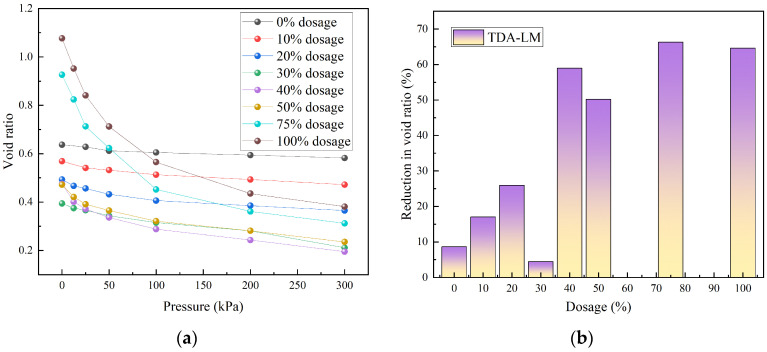
Variation pattern of void ratio of TDA-LM: (**a**) relationship between void ratio and pressure; (**b**) reduction in void ratio.

Figure 5 illustrates the variation in the void ratio (*e*) of tire-derived aggregate–loess mixtures (TDA-LMs) with pressure (*p*) under different tire particle contents. From Figure 5, the void ratio exhibited a significant negative correlation with applied pressure. Based on the morphological features of the *e*–*p* curves, the samples can be categorized into three distinct groups: (1) Low admixture group (0–20%): including pure loess and mixtures with 10–20% tire particles. The *e*–*p* curves in this group display relatively gentle slopes, with a reduction in void ratio ranging from 8.63% to 17.05% under 300 kPa compared to the initial void ratio. This indicated that the compressive behavior at low blending ratios was primarily controlled by the loess matrix; (2) Medium admixture group (30–50%): the compression behavior showed a clear two-stage characteristic. Under low pressures (0–100 kPa), the void ratio decreased sharply with a steep curve slope, whereas under high pressures (100–300 kPa), the compression curve flattened and converged toward trends similar to those of the low admixture group. The total reduction in void ratio at 300 kPa reached 25.96–58.95%, significantly higher than that of the low admixture group. These findings aligned with those reported by Li and Zhang [35,36], confirming the existence of a critical blending ratio around 30%; (3) High admixture group (75–100%): the *e*–*p* curves showed pronounced nonlinearity, with a transition point observed at approximately 100 kPa. Within the 0–100 kPa range, the curve exhibited marked nonlinear compression, reflecting initial rearrangement of the tire particle skeleton. Beyond 100 kPa, the compaction resistance increased, leading to a flatter segment of the curve. These patterns reflected a fundamental transition in the primary compaction mechanism within TDA–LM: from behavior dominated by the fine-grained soil matrix at low tire content to the restructuring and reinforcement of the granular skeleton at higher content. The 30–50% blending ratio represented a critical transition zone where both mechanisms coexisted. These results provide a theoretical basis for optimizing engineering design through admixture selection.

**Figure 6:**
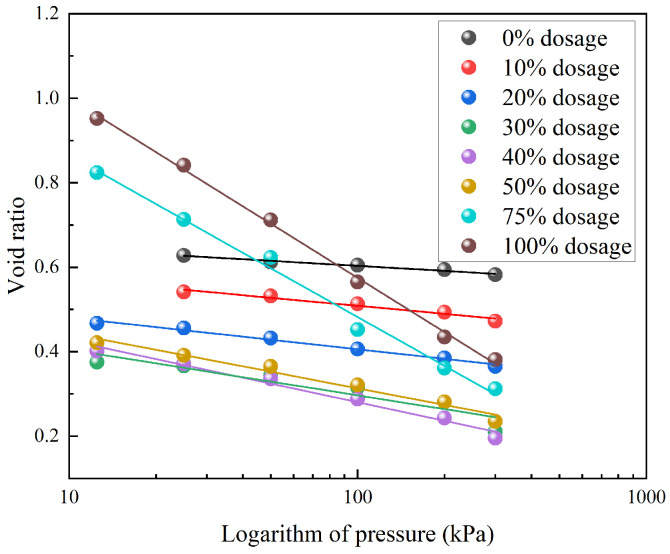
*e*-lg *p* curves of TDA-LM with varying mix proportions.

Figure 6 shows the relationship between the void ratio (*e*) and the logarithm of pressure (lg *p*) for TDA-LM with different mix proportions, and Table 4 summarizes the corresponding linear regression results. All specimens exhibited highly linear *e*–lg *p* relationships, with an average correlation coefficient (*R*^2^) of 0.968, confirming that semi-logarithmic coordinates suitably characterized the compression behavior of TDA-LM. A discrepancy of approximately 10% was observed between the theoretical mechanical data and the experimental data. The errors primarily arose from material heterogeneity and deviations in initial sample conditions. Further, boundary effects during loading caused the actual stress state to differ from the theoretical idealization. Based on the magnitudes of the compression indices, the specimens can be classified into three groups: (1) Low tire content (0–20%): displayed gentle slopes ranging from −0.039 to −0.075, indicating low compressibility; (2) Medium tire content (30–50%): showed steeper slopes between −0.108 and −0.146, reflecting significantly increased compressibility; (3) High tire content (75–100%): exhibited the steepest slopes, from −0.383 to −0.427, revealing high compression sensitivity. The increase in the absolute slope values with tire content provided direct evidence of the evolution in compression modulus. Specifically, as the tire particle content rose, the mechanical behavior transitioned from being dominated by the rigid loess matrix at low contents to being controlled by the compressible granular skeleton at high contents. These findings establish a quantitative basis for predicting settlement in engineering applications under various mix proportions.

**Figure 7:**
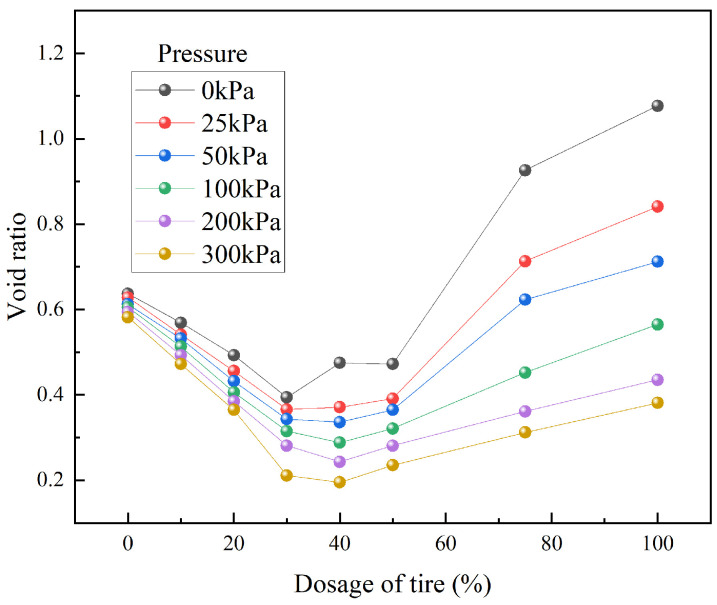
Effect of different proportions of waste tire particles on the void ratio of loess.

Figure 7 illustrates the influence of waste tire particle content on the void ratio of loess. From Figure 7, the void ratio of TDA-LM initially decreased and then increased with rising tire particle content, a trend attributable to changes in the contact state between coarse and fine particles within the mixture. When the applied pressure was less than or greater than 100 kPa, the boundary blending ratios corresponding to the minimum void ratio were 30% and 40%, respectively. The critical blending ratio fundamentally represented the optimal gradation between coarse and fine particles, while the critical pressure quantified the macroscopic stiffness threshold of the composite material under this optimal state. Further, specimens with 30% and 40% tire content reached their minimum void ratios under compressive stresses of 50 kPa and 100 kPa, respectively. Thus, the 30–40% range constituted the critical blending ratio interval for TDA-LM, accompanied by a threshold pressure range of 50–100 kPa. Within this parameter range, TDA-LM can fully utilize the synergistic effect between coarse and fine particles to achieve optimal deformation resistance.

### 3.2. Effect of Different Proportions of Waste Tire Particles on the Compression Coefficient of Loess

**Figure 8:**
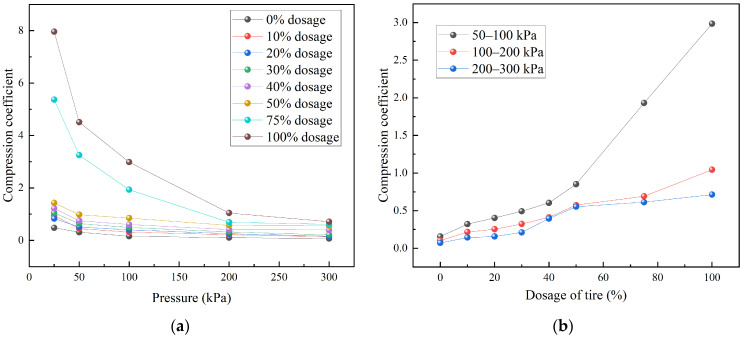
Variation pattern of compression coefficient of TDA-LM: (**a**) relationship between compression coefficient and pressure; (**b**) relationship between compression coefficient and the level of tire particles dosage.

Figure 8 illustrates the relationship between the compression coefficient and pressure for TDA–LM with various admixture ratios. From Figure 8a, the compression coefficient decreased gradually with increasing pressure. The compressibility of specimens containing 75% to 100% waste tire particles was significantly higher than that of pure loess and TDA–LM with 10% to 50% tire content. A distinct two-stage compression behavior was observed at a critical pressure of 100 kPa: (1) below 100 kPa, a rapid decrease in compressibility indicated initial structural rearrangement; (2) above 100 kPa, the asymptotic trend suggested stabilization of the particle skeleton. At 100 kPa, TDA–LM with tire-derived aggregate (TDA) contents between 30% and 100% exhibited compression coefficients exceeding 0.5, categorizing them as highly compressible soils. In comparison, specimens with 0–20% TDA content showed coefficients ranging from 0.156 to 0.403, typical of medium-compressibility soils. At 300 kPa, pure loess maintained low compressibility, whereas pure tire mixtures (100% TDA–LM) remained highly compressible. Further, the compression coefficient exhibited an overall positive correlation with tire content, while the threshold dosage displayed stress-dependent behavior. Under low-stress conditions, the critical dosage occurred at 30%, shifting to 40% under medium-to-high stress levels. This behavior arose because lower pressures (<100 kPa) primarily overcame interparticle friction, while higher pressures (>100 kPa) disrupted the particle structure itself, resulting in differing compression responses. The dosage range of 30–40% corresponded to the transition from a “soil matrix-controlled” mechanism to a “particle skeleton-dominated” one. Therefore, for projects expecting post-construction pressures exceeding 100 kPa, it is recommended to limit the TDA content to ≤40% to ensure subgrade stability. In low-stress applications, such as shallow pavement layers, the content can be increased up to 30% to improve deformation compatibility.

### 3.3. Effect of Different Proportions of Waste Tire Particles on the Compression Modulus of Loess

**Figure 9:**
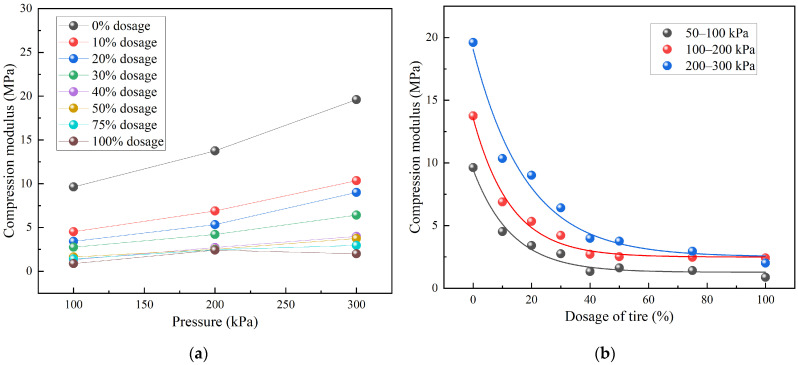
Variation pattern of compression modulus of TDA-LM: (**a**) relationship between compression modulus and pressure; (**b**) relationship between compression modulus and admixture content.

Figure 9 illustrates the variation in the compression modulus (*E_s_*) with pressure for TDA–LM with different admixture ratios. From Figure 9a, except for the pure tire sample, the *E_s_* values of all TDA–LM mixtures increased with pressure, demonstrating typical hardening behavior. The compression modulus of pure compacted loess was higher than that of any TDA–LM mixture across all pressure levels. In contrast, the pure tire specimen exhibited an anomalous softening response, with a 10% decrease in modulus within the 200–300 kPa range, which is attributed to the saturation of polymer chain orientation and the collapse of internal voids. Further, the sensitivity of the compression modulus to pressure decreased in the following order: pure compacted loess > TDA–LM with 10–30% tire content > TDA–LM with 40–100% tire content.

From Figure 9b, the compression modulus of TDA–LM was negatively correlated with tire content under all pressure levels. A distinct threshold was identified at approximately 40% tire content: below this value, the compression modulus was highly sensitive to changes in tire content, whereas above it, the modulus stabilized. This transition corresponded to the percolation threshold at which tire particles shifted from a dispersed phase to a continuous skeleton, dominating the mechanical behavior of the mixture. Table 5 presents the regression equations describing the compressive modulus of TDA–LM for different mix ratios. These equations indicated that the compression modulus followed an exponential decay function with increasing tire content across all pressure levels, reflecting the progressively disruptive effect of tire particles on the native soil structure. The high *R*^2^ values (ranging from 0.978 to 0.988) confirmed the excellent fit of the model to the experimental data. These equations allowed the prediction of *E_s_* for arbitrary tire contents under various pressures. For applications where modulus is critical (e.g., subgrade surface layers), it is recommended to limit the tire content to ≤30%. In addition, pure tire fillers should be avoided in high-pressure zones (>200 kPa) due to their inadequate load-bearing capacity.

### 3.4. Effect of Different Proportions of Waste Tire Particles on the Volume Compression Coefficient of Loess

**Figure 10:**
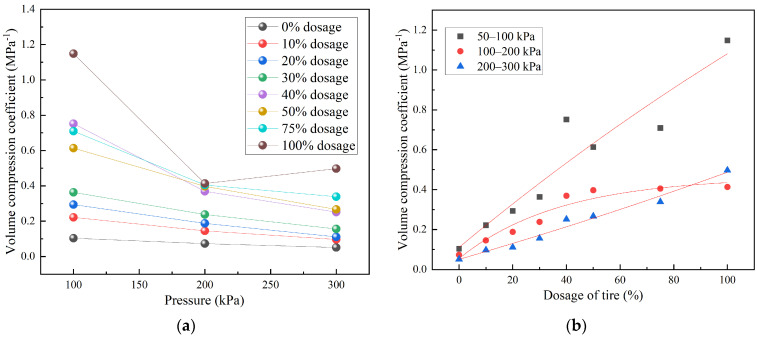
Variation pattern of volume compression coefficient of TDA-LM: (**a**) relationship between volume compression coefficient and pressure; (**b**) relationship between volume compression coefficient and the level of tire particles dosage.

Figure 10 shows the variation in the coefficient of volume compressibility (*m_v_*) with applied pressure for TDA–LM with different admixture ratios. From Figure 10a, all TDA–LM mixtures exhibited a negative correlation between *m_v_* and pressure, except for the pure tire sample. The pure tire specimen consistently showed higher *m_v_* values across all pressure levels compared to the TDA–LM blends. Moreover, the sensitivity of *m_v_* to pressure decreased in the following order: pure tire > TDA–LM with 40–75% tire content > pure loess and TDA–LM with 10–30% tire content. This trend can be attributed to the entropic elastic deformation mechanism inherent to rubber materials, which leads to higher volume compressibility in pure tire samples.

Figure 10b shows that the coefficient of volume compressibility (*m_v_*) of TDA–LM exhibits a positive exponential correlation with tire content under all pressure levels. The stress level was found to significantly influence the growth trend of *m_v_*. Within the medium-stress range (100–200 kPa), the relationship between *m_v_* and tire content displayed stronger nonlinearity, whereas this nonlinear behavior was less pronounced under both low- and high-stress conditions. This pattern can be attributed to the fact that the medium-stress interval corresponds to the critical state of soil–rubber interfacial slippage. In contrast, under high pressure, particle rearrangement was largely complete, leading to a more stabilized mechanical response. The regression equation for the *m_v_* of TDA–LM at various tire contents is provided in Table 6. These equations indicated an exponential growth relationship between *m_v_* and tire content across all pressure levels, with *R*^2^ values ranging from 0.901 to 0.979. Based on the regression models in Table 6, the volume compressibility of TDA–LM can be predicted for arbitrary admixture ratios and under different pressure conditions.

### 3.5. Stress–Strain Relationship of TDA-LM Under Cyclic Loading and Unloading

**Figure 11:**
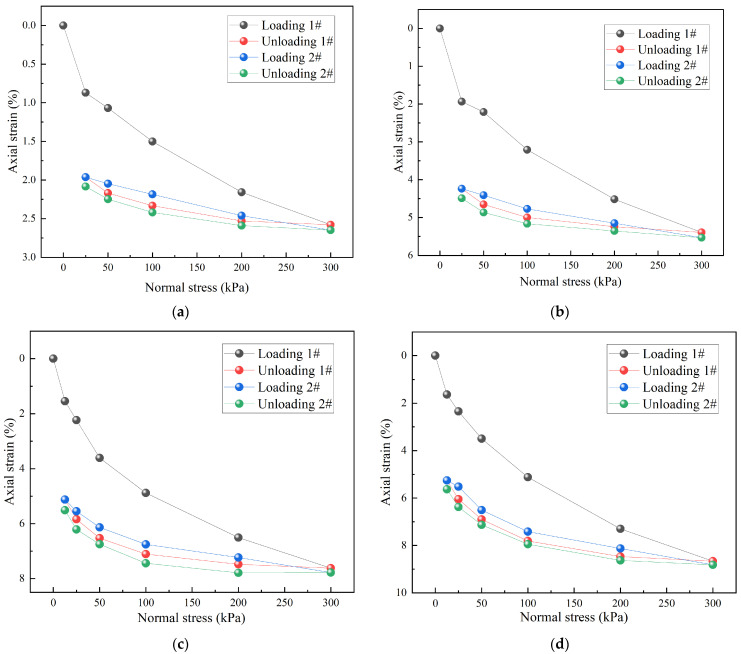
Stress–strain curves of TDA-LM with different dosages of tire particles: (**a**) 0% dosage-pure loess; (**b**) 10% dosage; (**c**) 20% dosage; (**d**) 30% dosage.

**Figure 12:**
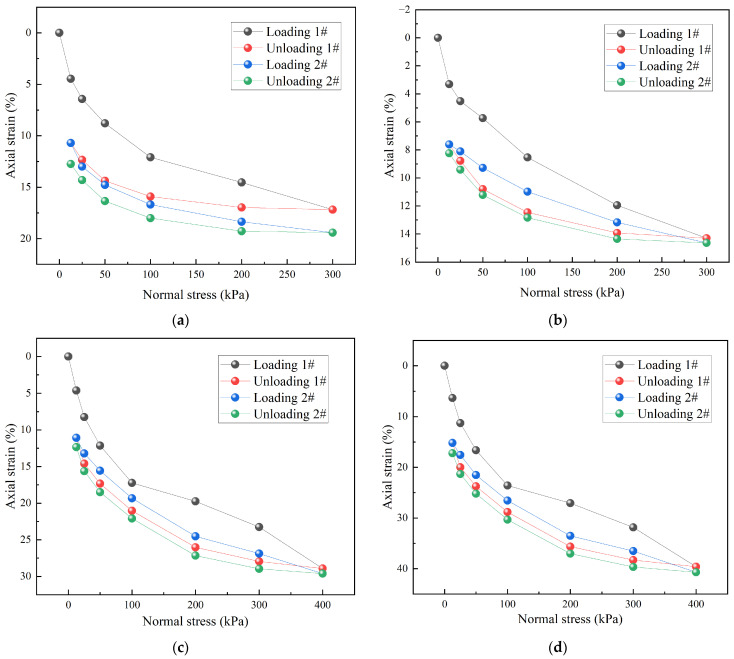
Stress–strain curves of TDA-LM with different dosages of tire particles: (**a**) 40% dosage; (**b**) 50% dosage; (**c**) 75% dosage; (**d**) 100% dosage.

To reveal the deformation mechanism of TDA-LM under cyclic loading and unloading conditions, the stress–strain response characteristics within the 0–100% dosage range were systematically studied (see Figure 11 and Figure 12). Although pure loess and 10% TDA-LM exhibited similar cyclic curve shapes, the latter showed more significant strain accumulation, which stemmed from the additional interfacial slip effect introduced by tire particles. The 10% TDA-LM mixture exhibited a 117% increase in strain increment during the second loading cycle (0.26%) compared to pure loess (0.12%). This indicated that the primary particle–soil interface restructuring was completed during the initial loading phase. The elastic strains of TDA-LM with 20% and 30% dosages were 2.49% and 3.41%, respectively. This difference was primarily attributed to the fact that at 30% dosage, the particle contact density reached the pre-percolation threshold critical state, thereby enhancing elastic energy storage capacity. This transition in strain energy distribution mechanism signified the material’s evolution from soil-dominated plastic deformation to a composite particle–soil elastoplastic behavior.

Figure 12 presents the stress–strain behavior of TDA–LM with dosages ranging from 40% to 100% under cyclic loading–unloading conditions. Although the 40% and 50% dosage specimens exhibited similar loading–unloading paths, the 50% mixture showed significantly improved recoverable strain characteristics. Under identical stress conditions, the irrecoverable strain between the first and second cycles differed by 2.04%. The high-dosage group (75–100%) developed substantial strains of 8.25–11.28% even under low stress (25 kPa), reaching 28.92–39.61% at 300 kPa [42]. These values aligned closely with the range of 26–33.6% reported by Masad et al. (1996) [42], with observed variations mainly attributable to the constraint effect of the loess matrix and specimen size effects. Notably, strain increments in all specimens occurred predominantly during the initial loading phase (contribution rate > 85%) and showed a positive exponential correlation with dosage content. The strain behavior of the 40% TDA–LM confirmed force-chain restructuring under optimal particle–matrix compatibility, providing a theoretical basis for determining the critical dosage in engineering applications. At Figure 12c (for 75% dosage of tire particles) and 12d (for 100% dosage of tire particles), for the very first time in the experimental part of the paper load goes to 400 kPa. This is because at higher dosages (75–100%), the tire particles form a continuous and dominant elastic skeleton, enabling the material to withstand higher loads without failure. Consequently, the experimental design required the application of a greater load (400 kPa) to investigate its critical state or failure mechanism of tire particles.

**Figure 13:**
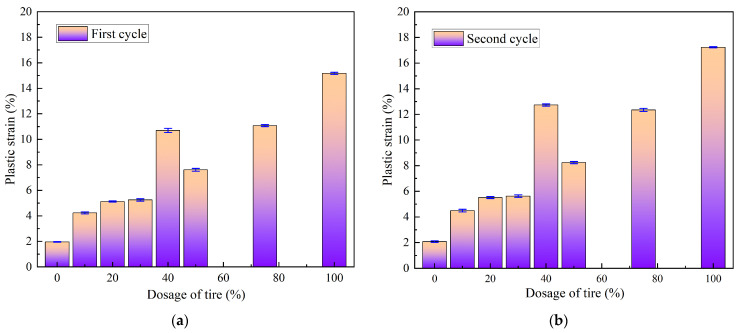
Plastic strain under different loading and unloading paths: (**a**) first cycle; (**b**) second cycle.

Figure 13 illustrates the irrecoverable strains (i.e., plastic strains) under various loading–unloading paths. The mean and standard deviation of the plastic strain data for different loading and unloading paths are shown in Table 7. From Figure 13 and Table 7, plastic strain, *ε_p_*, generally increased with dosage content. However, the 40% dosage mixture exhibited anomalously high plastic deformation. The plastic strain was lowest in pure loess (1.96%) and highest in pure waste tires (17.23%). Additionally, the plastic strain after the second cycle was, on average, 0.89% higher than that after the first cycle. The largest differences between the first and second cycles were observed in the 40% dosage and pure tire samples, with values of 2.04% and 2.06%, respectively. This behavior can be attributed to the 40% admixture representing the overspill threshold, where the soil–rubber interface area is maximized, leading to significant energy dissipation through friction. Therefore, in subgrades experiencing repeated loading, isolation layers are recommended in zones with 40% dosage content, while pure tire fillers should be restricted to non-load-bearing cushion applications.

**Figure 14:**
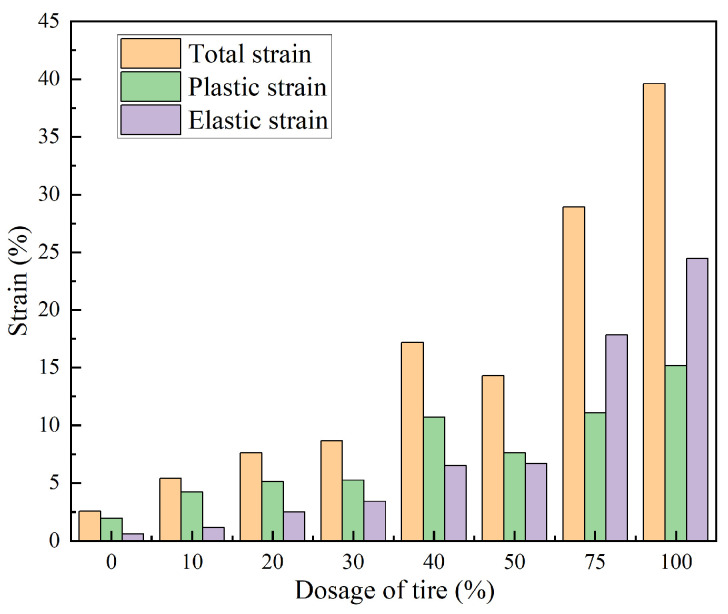
Elastoplastic strains of TDA-LM with varying dosages of tire particles.

Figure 14 shows the strain characteristics of TDA–LM with different dosage contents under maximum loading conditions, illustrating the evolution of total strain (*ε_t_*), plastic strain, *ε_p_*, and elastic strain, *ε_e_*, with increasing tire content. All three strain components increased with dosage content. Specimens with 30% and 40% dosage showed significant strain amplification at 300 kPa, while the 40–50% dosage range exhibited an anomalous strain reduction. This behavior stemmed from the percolation threshold reached at 40% dosage, where the particle–matrix system attained optimal compaction density and developed a stable force-chain network, thereby maximizing deformation resistance. At the critical dosage of 40%, the deformation resistance of the TDA–LM mixture peaked locally. Specifically, it exhibited a 50.87% improvement in plastic strain resistance compared to the 30% dosage mixture and a 28.86% improvement over the 50% dosage mixture. Meanwhile, the elastic strain of the 40% tire chip mixture was 3% lower than that of the 50% TDA–LM blend. Thus, for loess subgrades under medium to high stress conditions, a tire chip dosage of 40% is recommended as optimal. This dosage results in the highest resistance to plastic strain in the modified loess subgrade material, effectively curbing the accumulation of irrecoverable deformation under repeated traffic loads and significantly extending the service life of the subgrade. Additionally, the stable skeleton formed at this dosage helps distribute stress efficiently and reduces stiffness degradation, thereby ensuring sustained bearing capacity over the long term.

In terms of strain composition, all specimens except those with 75–100% dosage were characterized by *ε_p_* > *ε_e_*, indicating a deformation mechanism dominated by soil plasticity in the low-dosage regime. In contrast, specimens with 75–100% tire content displayed a strain reversal (*ε_e_* > *ε_p_*), confirming hyperelastic behavior resulting from a continuous rubber-phase skeleton. This shift in strain characteristics reflected a fundamental change in the material’s internal energy dissipation mechanism. For engineering applications of TDA–LM, the critical dosage of 40% served as a key reference for optimizing mechanical performance.

## 4. Discussion

Discussion centered on three primary themes: (1) the underlying mechanisms for the high *R*^2^ values; (2) the core findings of this study; and (3) the potential environmental impacts of tire particle-modified loess.

(1) The high *R*^2^ values of our predictive models stem from three key reasons: First, the models are physics-based, accurately capturing the fundamental phase transition from a soil-dominated to a rubber-dominated skeleton. Second, the mathematical forms of the models were chosen to align perfectly with the observed mechanical responses. Finally, the systematic experimental data, covering the full range of blend ratios and pressures, provided a solid foundation for robust parameter fitting. Therefore, the high accuracy is not coincidental but a result of correctly capturing the underlying mechanics.

(2) Beyond enhancing the mechanical properties of soil, a sufficient incorporation of waste tire shreds induces a fundamental change in the material’s load-bearing mechanism. In contrast to existing literature, this research provides a systematic exposition of the critical state characteristics and their evolution mechanisms in tire shred–loess composites under multiple stress levels. Predictive models are established for the compression modulus and volume compression coefficient, accounting for the coupling between stress level and tire content. The distribution patterns and evolutionary differences in elastoplastic deformation during cyclic loading-unloading are elucidated, thereby advancing the theoretical model’s predictive capability for practical engineering scenarios. Additionally, to address the absence of a standardized method for determining the critical content ratio, the optimal ratio for loess subgrade improvement is identified, along with its evolution with stress levels, supplying essential theoretical and technical parameters for relevant engineering design and construction. However, this study has not investigated the microstructure of the improved loess with waste tire chips. To address this limitation, future work will employ microscopic characterization techniques, such as scanning electron microscopy (SEM) and computed tomography (CT), to examine the microstructural evolution of loess and rubber skeleton restructuring.

(3) Although the improvement in mechanical properties of loess through tire chip modification is established, a thorough sustainability assessment requires critical examination of its concomitant environmental impacts. The incorporation of tire particles introduces risks of physical abrasion, chemical oxidation, and slow biodegradation, leading to the formation of microplastics and the leaching of chemical additives. The associated environmental risks are threefold: firstly, the migration of microplastics, where micron- and nano-scale particles from degradation could permeate soils and groundwater via seepage; secondly, the leaching of chemical constituents, including organic pollutants and heavy metals, which occurs gradually over the long term; and thirdly, potential ecological disruption, wherein these leached chemicals could alter soil microbial communities and impart long-term adverse effects on soil health. Subsequent research will therefore focus on systematic leaching tests and long-term field monitoring to enable a comprehensive sustainability evaluation of this technique.

## 5. Conclusions

This study presents a systematic investigation of the compression deformation characteristics of TDA-LM through comprehensive laboratory testing. The key findings can be summarized as follows:

(1) Compression behavior analysis reveals that high-dosage TDA-LM demonstrates comparable compression performance to pure tire specimens, while medium- and low-dosage mixtures exhibit behavior similar to conventional silt. Two critical dosage thresholds are identified, 30% and 40%, which demarcate distinct deformation responses below and above 100 kPa loading pressure, respectively.

(2) The compression coefficient shows significant dependence on both applied pressure (negative correlation) and tire content (positive correlation). The optimal dosage is stress-dependent, with 30% being preferable under low-stress conditions (<100 kPa) and 40% under medium-high stress conditions. For engineering applications, dosage should be constrained to ≤40% in high-pressure zones (>100 kPa) to ensure stability, while ≤30% is recommended for low-stress environments to enhance deformation performance.

(3) The variation pattern of TDA-LM’s compression coefficient with pressure and dosage content was revealed. A predictive model has been developed to estimate both the compression modulus and volumetric compression coefficient across various pressure levels and arbitrary dosage contents. Notably, medium-low dosage TDA-LM exhibits hardening behavior, contrasting with the 10% modulus softening observed in pure tire specimens at 200–300 kPa. The compression modulus sensitivity follows a distinct hierarchy: pure loess > 10–30% TDA-LM > 40–100% TDA-LM. Practical recommendations include limiting dosage to ≤30% in modulus-sensitive applications and avoiding pure tire filler in scenarios exceeding 200 kPa.

(4) Cyclic loading tests uncover the dual deformation mechanisms in TDA-LM: soil-dominated plastic deformation at low dosages versus rubber-dominated elastic behavior at high dosages. Over 85% of tire–soil interface restructuring occurs during the first loading. The 40% dosage anomaly confirms optimal force-chain restructuring, establishing critical dosage criteria for engineering applications.

## Figures and Tables

**Table 1:** Physical and mechanical properties of loess on the Yan’an Expressway.

Item	Water Content (%)	Dry Density (g/cm^3^)	Specific Gravity	Void Ratio	Liquid Limit (%)	Plastic Limit (%)	Plastic Index
Mean	12.12	1.42	2.69	0.98	28.1	18.6	8.5
Loess range	7–18.2	1.19–1.49	-	0.78–1.22	25.6–28	17.1–19.3	7.2–9.9

**Table 2:** Physical properties of samples with different proportions of waste tire particles.

Waste Tire Content (%)	Initial Water Content (%)	Initial Density (g/cm^3^)	Initial Void Ratio	Specific Gravity
0	15.2	1.92	0.66	2.69
10	13.6	1.73	0.59	2.37
20	13.8	1.65	0.52	2.12
30	15.1	1.61	0.41	1.93
40	13.4	1.30	0.59	1.75
50	13.1	1.27	0.52	1.61
75	8.5	0.82	0.75	1.28
100	0.72	0.56	1.18	1.15

**Table 3:** Summary of compression test results.

Waste Tire Content (%)	Void Ratio			Compression Coefficient (MPa^−1^)			Compression Modulus (MPa)		
	50–100 kPa	100–200 kPa	200–300 kPa	50–100 kPa	100–200 kPa	200–300 kPa	50–100 kPa	100–200 kPa	200–300 kPa
0	0.605	0.594	0.582	0.156	0.103	0.071	9.63	13.75	19.61
10	0.513	0.493	0.472	0.320	0.215	0.142	4.52	6.89	10.35
20	0.406	0.385	0.365	0.403	0.253	0.156	3.41	5.33	9.01
30	0.315	0.281	0.211	0.492	0.322	0.211	2.75	4.21	6.42
40	0.288	0.243	0.195	0.603	0.411	0.395	1.33	2.71	3.98
50	0.321	0.281	0.235	0.851	0.573	0.552	1.63	2.52	3.75
75	0.452	0.361	0.312	1.931	0.691	0.613	1.41	2.47	2.952
100	0.565	0.435	0.381	2.985	1.043	0.713	0.871	2.42	2.01

**Table 4:** Regression equations between void ratio (*e*) and logarithmic pressure (lg *p*) for TDA-LM with varying mix proportions.

Content (%)	Regression Equations	*R* ^2^
0	*e* = 0.682 − 0.039lg *p*	0.978
10	*e* = 0.635 − 0.063lg *p*	0.961
20	*e* = 0.560 − 0.075lg *p*	0.986
30	*e* = 0.513 − 0.108lg *p*	0.875
40	*e* = 0.573 − 0.146lg *p*	0.980
50	*e* = 0.574 − 0.130lg *p*	0.974
75	*e* = 1.248 − 0.383lg *p*	0.992
100	*e* = 1.428 − 0.427lg *p*	0.998

**Table 5:** Regression equations for compression modulus (*Es*) of TDA-LM with varying tire contents.

Pressure Range (kPa)	Regression Equations	*R* ^2^
50–100	$y=2.510+16.561\exp\left(-0.055x\right)$	0.978
100–200	$y=2.490+11.087\exp\left(-0.078x\right)$	0.988
200–300	$y=1.279+8.187\exp\left(-0.075x\right)$	0.979

**Table 6:** Regression equations for the volume compression coefficient of TDA-LM with varying tire contents.

Pressure Range (kPa)	Regression Equations	*R* ^2^
50–100	$y=3.902-3.792\exp\left(-0.003x\right)$	0.901
100–200	$y=0.464-0.409\exp\left(-0.027x\right)$	0.939
200–300	$y=-1.672+1.723\exp\left(0.002x\right)$	0.979

**Table 7:** Statistical data of plastic strain under different loading and unloading paths.

Item	Dosage of Tire (%)	Plastic Strain (%)			Mean (%)	Standard Deviation
		Test 1	Test 2	Test 3		
First cycle	0	1.951	1.989	1.946	1.962	0.02352
	10	4.156	4.315	4.24	4.237	0.07954
	20	5.085	5.168	5.125	5.126	0.04151
	30	5.156	5.332	5.28	5.256	0.09042
	40	10.521	10.723	10.853	10.699	0.1673
	50	7.55	7.73	7.55	7.61	0.10392
	75	10.987	11.123	11.121	11.077	0.07795
	100	15.085	15.224	15.213	15.174	0.07727
Second cycle	0	2.125	2.013	2.111	2.083	0.06102
	10	4.521	4.382	4.573	4.492	0.09875
	20	5.43	5.59	5.51	5.51	0.08
	30	5.52	5.71	5.66	5.63	0.09849
	40	12.64	12.79	12.775	12.735	0.08261
	50	8.15	8.31	8.266	8.242	0.08266
	75	12.22	12.45	12.38	12.35	0.1179
	100	17.18	17.26	17.25	17.23	0.04359

## Data Availability

The original contributions presented in this study are included in the article. Further inquiries can be directed to the corresponding author.

## Abbreviations

The following abbreviations are used in this manuscript:

TDA-LM	Tire-derived aggregate–loess mixture
TDA-SM	Tire-derived aggregate–sand mixture
CRIA	China Rubber Industry Association
